# Hepatitis E Virus Infection in Pregnant Women

**DOI:** 10.3390/microorganisms14092105

**Published:** 2026-09-20

**Authors:** Zheng Wang, Yun-Sheng Ge, Min-Ming Wang, Yu-Lin Zhou, Gui-Ping Wen

**Affiliations:** Department of Central Laboratory, Fujian Key Clinical Specialty of Laboratory Medicine, Department of Obstetrics and Gynecology, Women and Children’s Hospital, School of Medicine, Xiamen University, Xiamen 361003, China

**Keywords:** hepatitis E virus, pregnant women, diagnosis, prevalence, clinical manifestations, mechanisms, treatment, prevention

## Abstract

Hepatitis E virus (HEV) is a major global public health concern, particularly in resource-limited regions. Approximately 12.5% of individuals worldwide have been exposed to HEV, and 15–110 million individuals have current or recent HEV infection. HEV poses an especially serious threat to pregnant women, in whom infection can progress to severe disease and is associated with a high case fatality rate, particularly during the third trimester. Moreover, maternal HEV infection can lead to various adverse outcomes in fetuses and/or neonates, as documented in both developing and developed countries. No HEV-specific antiviral therapy is approved for use during pregnancy, and pregnancy-specific evidence and recommendations for HEV vaccination remain limited. This narrative review summarizes the diagnosis, prevalence, clinical manifestations, mechanisms underlying severe disease, treatment, and prevention of HEV infection in this high-risk population. It aims to improve clinicians’ awareness of the potential impact of HEV infection on pregnant women and their fetuses/neonates, to promote public health literacy, and to accelerate future disease-control efforts.

## 1. Introduction

Hepatitis E virus (HEV) is a global health concern affecting both low- and middle-income countries and industrialized countries, and it is the most common cause of acute viral hepatitis (AVH) worldwide [1]. Approximately 12.5% of the global population have ever experienced HEV infection, and 15–110 million individuals have a current or recent infection [2]. The World Health Organization (WHO) estimated that HEV caused 19.47 million acute hepatitis E cases and 3450 deaths worldwide in 2021 [3]. HEV also has a high potential for zoonotic transmission, ranking sixth among 887 wildlife viruses in a risk assessment of animal-to-human spillover [4].

HEV is a single-stranded RNA virus classified within the family *Hepeviridae*. This family is divided into two subfamilies: *Orthohepevirinae*, which contains four genera (*Avihepevirus*, *Chirohepevirus*, *Paslahepevirus*, and *Rocahepevirus*), and *Parahepevirinae*, which comprises the single genus *Piscihepevirus* [5]. The species *Paslahepevirus balayani*, a member of the genus *Paslahepevirus*, encompasses eight genotypes. Four of these genotypes are most commonly associated with human HEV infection and exhibit distinct geographic distributions and prevalence patterns. HEV genotype 1 (HEV-1) and genotype 2 (HEV-2) circulate exclusively in humans and are associated with large outbreaks in developing countries, particularly in areas with poor sanitation [6]. HEV genotype 3 (HEV-3) and genotype 4 (HEV-4) are zoonotic, possess animal reservoirs, and cause sporadic cases worldwide [7]. In addition, a single human infection with HEV-7 has been documented in association with the consumption of camel meat and milk [8]. HEV-1 and HEV-2 are transmitted primarily via the fecal-oral route, whereas HEV-3 and HEV-4 are transmitted primarily through the foodborne route via consumption of raw or undercooked animal meat. Furthermore, HEV can be transmitted through blood transfusion [9,10,11] and vertical transmission [12,13,14,15,16,17]. Another possible route involves occupational exposure among individuals in close contact with infected animals [18]. The HEV genome is approximately 7.2 kb in length and contains three primary open reading frames (ORFs), with a fourth ORF observed in HEV-1 [19,20].

HEV infection is usually asymptomatic or self-limiting in the general population, with a relatively low mortality rate of 0.2–1% [21]. However, it poses a significant risk to pregnant women, especially in the third trimester. In this population, HEV infection may lead to severe clinical outcomes. Case-fatality rates ranging from 1.8% to 66.7% have been reported among HEV-infected pregnant women in endemic settings where HEV genotype 1 and genotype 2 predominate, although the infecting genotype was not confirmed in many of these studies [12,13,14,15,16,17,22,23,24,25,26,27,28,29,30,31,32,33,34,35,36,37,38,39,40,41,42,43,44,45,46,47,48,49,50,51,52,53,54]. Moreover, HEV infection is associated with adverse fetal outcomes, including miscarriage, stillbirth, preterm birth, low birth weight, and intrauterine fetal death [55]. To date, no HEV-specific antiviral therapy is available for pregnant women. Although an HEV vaccine has been approved, evidence regarding its efficacy and safety in pregnant women remains limited [56]. This narrative review summarizes current knowledge and ongoing research on HEV infection during pregnancy and highlights key areas warranting further investigation. It aims to enhance public awareness, particularly among healthcare providers, and to accelerate future disease control efforts.

## 2. Diagnosis of HEV Infection

**Figure 1:**
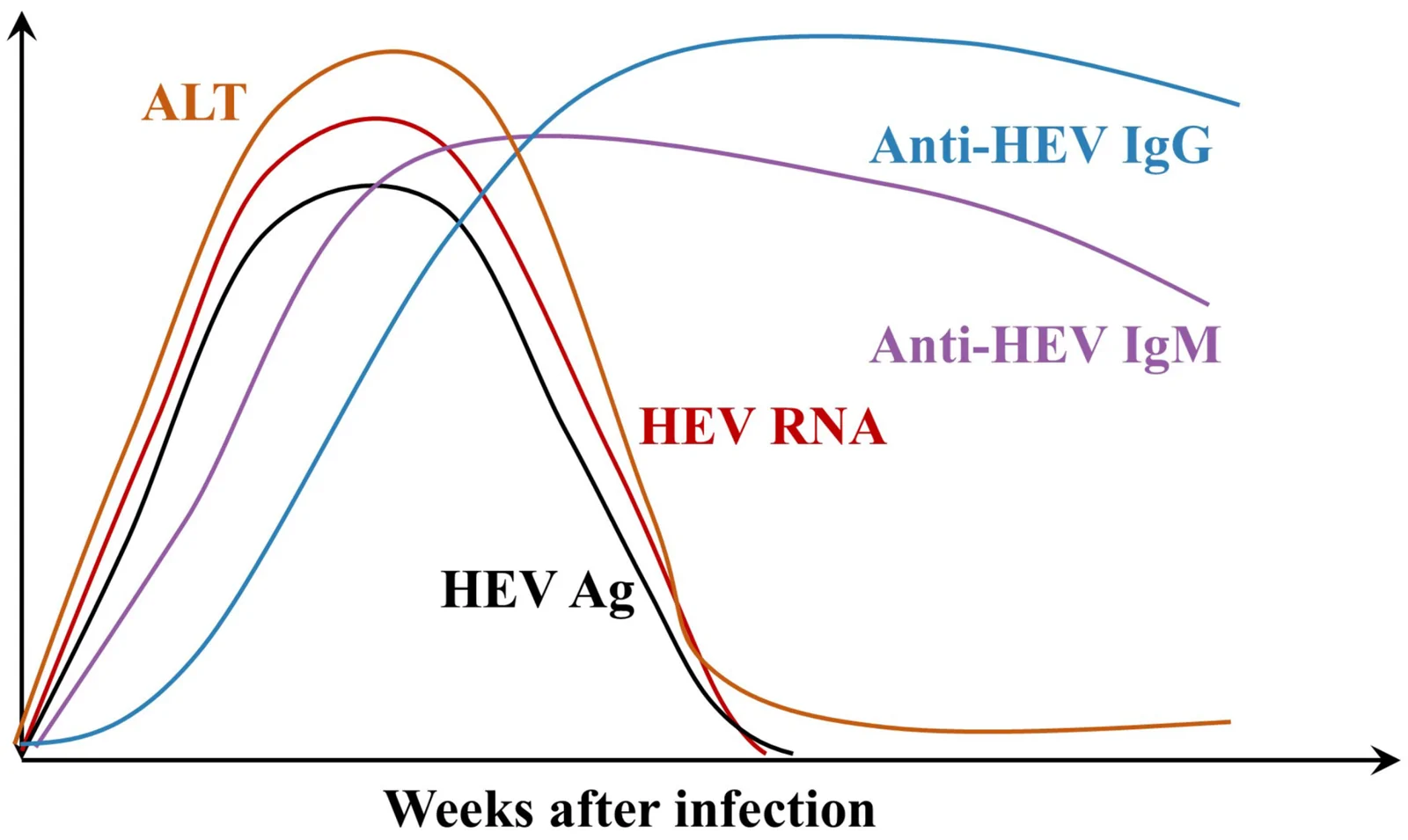
Diagnosis of HEV infection. Typical evolution of the HEV-related makers and levels of alanine aminotransferase (ALT) levels during HEV infection. HEV Ag: HEV antigen.

The clinical features of HEV infection are often nonspecific [57]. In pregnant individuals presenting with liver dysfunction, it is essential to distinguish among pregnancy-related liver diseases, HEV infection, and other hepatic injuries. HELLP syndrome, a severe preeclampsia-related complication, is characterized by hemolysis, low platelets, and elevated liver enzymes [58,59]. Acute fatty liver of pregnancy occurs most commonly in the late third trimester; clinical presentations range from nausea and abdominal pain to hepatic encephalopathy and jaundice, accompanied by hyperammonemia and coagulopathy [58,59]. Intrahepatic cholestasis of pregnancy, the most common gestational liver disease, is characterized by pruritus, and the diagnosis can be confirmed by elevated serum bile acids levels [58,59]. Diagnosis of drug-induced liver injury relies on a clear temporal association between drug exposure and abnormal liver-function tests, together with the exclusion of alternative causes of hepatocellular injury [59]. Viral hepatitis caused by hepatitis A virus (HAV), hepatitis B virus (HBV), hepatitis C virus (HCV), cytomegalovirus (CMV), Epstein–Barr virus (EBV), and HEV can be identified using corresponding serological and molecular assays. Laboratory diagnosis of HEV relies primarily on four markers: HEV RNA, HEV antigen, anti-HEV IgM, and anti-HEV IgG (Figure 1).

HEV RNA is considered the gold standard for diagnosing current HEV infection, providing a highly sensitive and specific approach, particularly in immunosuppressed individuals. HEV RNA can be detected in blood, stool, and urine approximately three weeks after infection [60,61,62,63]. Real-time reverse transcription-polymerase chain reaction (RT-PCR) represents the well-established clinical technique [64,65]. Notably, real-time RT-PCR assays targeting ORF3 have shown superior sensitivity compared with those targeting ORF2 [66]; consequently, most commercial qPCR kits target ORF3 [67]. Several emerging nucleic-acid-based diagnostic technologies have been developed for HEV detection, including loop-mediated isothermal amplification (LAMP) assays [68,69], recombinase polymerase amplification (RPA) assays [70,71,72], and a Cas13a-crRNA-based assay [73]. LAMP assays have been evaluated using both human clinical specimens and porcine samples [68,69]. Most reported RPA assays have been validated using raw pork liver and rabbit samples, with one study also including human samples [70,71,72]. In addition, a Cas13a-crRNA-based assay provides an additional option for HEV RNA detection [73]. At present, these isothermal and CRISPR-based approaches remain largely experimental and have not been routinely adopted for clinical diagnosis of HEV infection. HEV RNA detection is generally technically complex, time-consuming, and costly, which limits its widespread application, especially in resource-limited settings.

HEV antigen detection has emerged as a valuable tool for diagnosing current HEV infection [61,74]. Its primary target is the free secreted form of the ORF2 protein (ORF2^S^), which is the predominant form in serum and is not associated with the viral genome or virions [75,76,77]. Similar to HEV RNA, HEV antigen is detectable in feces, blood, and urine [61,63,74]. Notably, HEV antigen levels in urine and the corresponding diagnostic sensitivity have been reported to be higher than those in serum [61]. HEV antigen detection based on enzyme-linked immunosorbent assays (ELISAs) has demonstrated high sensitivity for diagnosing hepatitis E [62,76,78,79], and HEV antigen levels can also predict the outcomes of HEV infection [74]. In addition, a fluorescent microbead-based immunoassay has been developed as an alternative approach for HEV antigen detection [80]. Recently, a digital biosensor using graphene field-effect transistors functionalized with llama-derived nanobodies was developed to detect HEV antigen, achieving a limit of detection in the nanomolar range [81]. HEV antigen detection is recommended for resource-limited regions and clinical settings that lack molecular diagnostic tools [74].

Anti-HEV antibodies are the most widely used clinical diagnostic markers for HEV infection. Anti-HEV IgM assays are regarded as a routine diagnostic method, with the presence of anti-HEV IgM indicating recent infection. Anti-HEV IgM typically develops approximately four weeks after infection or shortly after the onset of clinical symptoms [82,83,84]. Previous studies have shown that the duration of anti-HEV IgM positivity appears to be considerably longer than that of HEV viremia, antigenemia, and biochemical marker abnormalities [62,79,84]. Anti-HEV IgM can persist for up to 8 months after alanine aminotransferase levels return to normal [62,79], and one study demonstrated that it remained detectable after 14 months in 25% of cases [84]. Anti-HEV IgG, regarded as a marker of prior exposure, appears a few days after anti-HEV IgM but usually persists for years rather than months [57]. Various formats have been developed to detect anti-HEV IgM or IgG, including enzyme immunoassays and rapid immunochromatographic assays, among which ELISA is the most frequently used format [57]. HEV has been difficult to culture in vitro owing to its slow replication, low yield, and the absence of efficient cell culture systems for all HEV genotypes [85,86,87], which poses significant challenges for virus isolation and precludes the use of natural viruses for anti-HEV antibody assays. Both recombinant proteins and synthetic peptides have been used as diagnostic antigens in anti-HEV antibody assays [57,88]. Generally, assays using recombinant proteins as diagnostic antigens exhibit higher sensitivity than those using synthetic peptides [57,89,90,91]. Numerous commercial and in-house assays have been developed, and their diagnostic performance characteristics vary considerably [92,93]. Of note, anti-HEV antibodies may be undetectable in some individuals, including both immunocompetent and immunosuppressed patients [94,95,96,97,98].

Overall, HEV RNA detection remains the most reliable method for confirming active HEV infection, whereas serological tests are useful but may be limited by the timing of sample collection and antibody persistence.

## 3. Prevalence of HEV Infection

The earliest documented evidence of HEV infection in pregnant women dates back to the 1978 non-A, non-B hepatitis outbreak in India [99]. Since 1996, numerous studies have reported the prevalence of HEV infection in pregnant women worldwide, revealing considerable geographic variation (PubMed was searched using the keywords “HEV”/“hepatitis E virus”/“hepatitis E,” “pregnant”/“pregnancy,” and “prevalence,” with the last search conducted on 25 May 2026; studies were included if they reported original data with sufficient information available in the abstract or main text, whereas reviews and studies with insufficient data were excluded) (Table 1) [17,26,40,43,46,47,52,100,101,102,103,104,105,106,107,108,109,110,111,112,113,114,115,116,117,118,119,120,121,122,123,124,125,126,127,128,129,130,131,132,133,134,135,136,137,138,139,140,141,142,143,144,145,146,147,148,149,150,151,152,153,154,155,156,157,158,159,160,161,162]. Most of these studies have focused on seroprevalence (anti-HEV IgM, anti-HEV IgG, and/or anti-HEV total Abs). Seroprevalence among pregnant women was relatively low in Argentina [100], Cameroon [106,107,108], Côte d’Ivoire [118], France [126], Iran [138,139,140,141], Italy [142], Lebanon [143], Mexico [144,145], Senegal [152], South Africa [153], and Spain [154,155], whereas it was high in Egypt [120,121,122,123], Ethiopia [124,125], India [40,134,135,136], Nigeria [43,146,147,149], and Pakistan [46,47,150]. The anti-HEV IgM positivity rate exceeded 10% in Bangladesh (38.5%) [26], India (15.4–18.6%) [40,135], Pakistan (13.3–15.6%) [46,150], Sudan (10.3%) [52], and Thailand (11.8%) [157]. Among pregnant women in China, the prevalences of anti-HEV IgG, anti-HEV IgM, and HEV RNA were 6.3–16.6%, 0.0–4.4%, and 0.0–0.5%, respectively [109,110,111,112,113,114,115,116,117]. HEV RNA prevalence was <1% in most countries [100,101,102,104,109,111,114,115,116,117,132,133,139,153,154,155,162], whereas considerably higher HEV RNA prevalence was documented in India (12.8–29.2%) [40,135], Sudan (5.1%) [52], and the United Arab Emirates (6.0%) [17]. HEV antigen positivity was assessed in only two countries: China (0.3%) [116] and Ghana (1.0%) [132]. Information on HEV genotype among infected pregnant women has been reported in only a limited number of studies (Table 1) [40,107,108,112,114,147,161]. Specifically, HEV-1 has been detected in infected pregnant women in India [40] and Nigeria [147]. HEV-3 has been identified in pregnant cases from Cameroon [107,108] and Vietnam [161], and HEV-4 infection has been documented among pregnant women in China [112,114]. Most studies on HEV infection among pregnant women have not provided genotyping information.

**Table 1:** The prevalence of HEV infection in pregnant women in different countries.

First Author, Year of Publication	Country	Sample Size (*n*)	Gestational Period (Trimesters)	Anti-HEV IgM (%)	Anti-HEV IgG (%)	Anti-HEV Total Antibodies (%)	HEV RNA (%)	HEV Antigen (%)	Genotypes ^a^	References
Tissera et al., 2020	Argentina	202	1st to 3rd	1.0	8.4	-	0.0 ^1^	-		[100]
Paul et al., 2020	Bangladesh	192	-	38.5	-	-	-	-		[26]
De Paschale et al., 2016	Benin	278	1st to 3rd	1.4	16.2	-	0.0 ^1^	-		[101]
Hardtke et al., 2018	Brazil	209	-	-	19.1	-	0.0 ^1^	-		[102]
Traore et al., 2012	Burkina Faso	189	-	-	11.6	-	-	-		[103]
Chhoung et al., 2025	Burkina Faso	491	-	2.5	18.6	-	0.0 ^1^	-		[104]
Mirzaev et al., 2024	Cambodia	1565	-	2.6	-	11.6	-	-		[105]
Modiyinji et al., 2019	Cameroon	183	-	14.8	4.9	-	-	-		[106]
Mbencho et al., 2025	Cameroon	87	-	0.0	5.7	-	2.3	-	3e	[107]
Mbencho et al., 2025	Cameroon	190	3rd	1.1	3.7	-	1.1	-	3e	[108]
Huang et al., 2013	China	293	1st to 3rd	1.4	10.2	-	0.0	-		[109]
Cong et al., 2015	China	990	1st to 3rd	2.6	16.2	-	-	-		[110]
Gu et al., 2015	China	497	2nd	0.6	11.1	-	0.0 ^1^	-		[111]
Huang et al., 2015	China	274	1st to 3rd	3.7	8.8	-	-	-	4	[112]
Huang et al., 2016	China	391	3rd	0.0	10.7	-	-	-		[113]
Li et al., 2020	China	946	1st to 3rd	4.4	16.6	-	0.1 ^1^	-	4a	[114]
Ma et al., 2021	China	3278	1st to 2nd	0.6	6.3	-	0.0 ^1^	-		[115]
Wen et al., 2023	China	4244	2nd	0.4	10.5	-	0.5	0.3		[116]
Qian et al., 2023	China	19,762	-	0.3	11.5	-	0.0 ^1^	-		[117]
Sevede et al., 2019	Côte d’Ivoire	200	-	-	1.5	-	-	-		[118]
Jelicic et al., 2022	Croatia	118	-	0.0	1.7	-	-	-		[119]
Amer et al., 1996	Egypt	18	-	-	-	55.6	-	-		[120]
Stoszek et al., 2006	Egypt	2428	2nd and 3rd	-	-	84.3	-	-		[121]
Gad et al., 2011	Egypt	116	-	-	58.6	-	-	-		[122]
El-Tras et al., 2013	Egypt	16	-	-	25.0	-	-	-		[123]
Abebe et al., 2017	Ethiopia	386	1st to 3rd	0.5	31.6	-	-	-		[124]
Niguse et al., 2018	Ethiopia	846	1st to 3rd	1.0	42.4	-	-	-		[125]
Renou et al., 2014	France	315	-	0.0	7.7	-	-	-		[126]
Caron et al., 2008	Gabon	840	-	-	14.2	-	-	-		[127]
Caron et al., 2012	Gabon	243	-	-	6.6	-	-	-		[128]
Zollkau et al., 2022	Germany	62	-	0.0	9.7	-	-	-		[129]
Adjei et al., 2009	Ghana	157	1st to 3rd	18.5	10.2	-	-	-		[130]
Obiri-Yeboah et al., 2018	Ghana	398	1st to 3rd	0.3	12.1	-	-	-		[131]
Bagulo et al., 2024	Ghana	1000	1st to 3rd	0.0	8.3	-	0.0 ^2^	1.00		[132]
Tejada-Strop et al., 2019	Haiti	1279	-	0.3	10.3	-	0.0 ^1^	-		[133]
Begum et al., 2009	India	300	2nd	-	33.7	-	-	-		[134]
Bharadia et al., 2011	India	236	1st to 3rd	18.6	10.3	-	29.2	-		[135]
Malhotra et al., 2020	India	39	1st to 3rd	15.4	-	-	12.8	-	1a	[40]
Singh et al., 2026	India	602	1st to 3rd	-	47.7	-	-	-		[136]
Surya et al., 2005	Indonesia	819	-	-	18.4	-	-	-		[137]
Rostamzadeh Khameneh et al., 2013	Iran	136	-	-	3.7	-	-	-		[138]
Farshadpour et al., 2018	Iran	1331	1st to 3rd	0.8	6.2	-	0.0 ^1^	-		[139]
Kenarkoohi et al., 2021	Iran	420	-	0.5	-	4.3	-	-		[140]
Sadeghi et al., 2021	Iran	247	1st to 3rd	-	0.8	-	-	-		[141]
La Fauci et al., 2017	Italy	352	1st to 2nd	-	-	3.4	-	-		[142]
Ismail et al., 2020	Lebanon	450	1st to 3rd	-	0.2	-	-	-		[143]
Alvarado-Esquivel et al., 2014	Mexico	439	1st to 3rd	-	5.7	-	-	-		[144]
Baptista-Gonzalez et al., 2017	Mexico	127	1st to 3rd	-	0.8	-	-	-		[145]
Junaid et al., 2014	Nigeria	108	-	0.9	41.7	-	-	-		[146]
Fowotade et al., 2020	Nigeria	230	1st to 3rd	4.8	17.0	-	1.3 ^1^		1	[147]
Okwara et al., 2021	Nigeria	200	2nd and 3rd	15.0	19.0	-	-	-		[43]
Osundare et al., 2021	Nigeria	160	1st to 3rd	0.6	-	6.3	-	-		[148]
Ehi Airiohuodion et al., 2022	Nigeria	200	1st to 3rd	-	31.5	-	-	-		[149]
Javed et al., 2017	Pakistan	135	1st to 3rd	15.6	-	-	-	-		[46]
Fatima et al., 2020	Pakistan	90	1st to 3rd	13.3	60.0	-	-	-		[150]
Aisha et al., 2024	Pakistan	500	1st to 3rd	-	21.0	-	-	-		[47]
Mesquita et al., 2013	Portugal	12	-	-	33.3	-	-	-		[151]
Diouara et al., 2022	Senegal	1227	-	0.5	7.4	-	-	-		[152]
Simani et al., 2022	South Africa	384	-	0.0	3.1	3.1	0.0 ^1^	-		[153]
Lindemann et al., 2010	Spain	1040	1st	0.7	3.7	-	0.0 ^1^	-		[154]
Buti et al., 2010	Spain	1517	-	0.0	5.4	-	0.0 ^1^	-		[155]
Elduma et al., 2014	Sudan	39	-	10.3	-	-	5.1 ^1^	-		[52]
Poovorawan et al., 1996	Thailand	178	-	-	9.0	-	-	-		[156]
Boonyai et al., 2021	Thailand	17	1st to 3rd	11.8	41.2	-	-	-		[157]
Ayouni et al., 2026	Tunisia	891	1st to 3rd	-	3.8	-	0.1 ^1^	-		[158]
Cevrioglu et al., 2004	Turkey	245	3rd	0.0	12.7	-	-	-		[159]
Oncu et al., 2006	Turkey	386	-	-	7.0	-	-	-		[160]
Kumar et al., 2001	United Arab Emirates	469	3rd	-	-	19.8	6.0 ^1^	-		[17]
Huy et al., 2021	Vietnam	183	3rd	2.2	7.7	-	2.2	-	3a	[161]
Thi Hong Van et al., 2025	Vietnam	528	3rd	2.5	24.1	-	0.0	-		[162]

^1^ HEV RNA detection was only performed in samples positive for anti-HEV antibodies (anti-HEV IgM, anti-HEV IgG, and/or total anti-HEV antibodies). ^2^ HEV RNA was performed in HEV antigen-positive samples. - indicates not reported. ^a^ The genotypes in the studied pregnant women. Lowercase letters represent subgenotypes.

The prevalence of HEV infection in pregnant women has been reported to increase with age in multiple studies [43,110,116,117,124,131,144,146,147,156,158,159,162]. Other associated factors included income level [146,159], rural residence [146,159], method of fecal disposal [43,146], contact with pigs [110], exposure to soil [110], and source of drinking water [43,146]. Collectively, these observations suggest that adequate hygiene practices and sanitary conditions may be important for maintaining a low HEV prevalence. Whether gravidity and parity are associated with HEV infection remains unclear, as previous studies have yielded inconsistent findings [117,124,131].

Taken together, the prevalence of HEV infection in pregnant women varies considerably by region, likely reflecting differences in sanitation, endemicity, and genotype distribution. Notably, diagnostic methods also influence reported prevalence, given the substantial inter-assay variability in both anti-HEV and HEV RNA detection.

## 4. Clinical Manifestations and Outcomes

Clinically, most HEV infections are asymptomatic or self-limiting, causing only mild symptoms. The proportion of symptomatic infections ranges from 5% to 30%, depending on the HEV genotype [163]. The clinical features of HEV infection cannot be distinguished clinically from those of other types of acute viral hepatitis; they include jaundice, fatigue, poor appetite, rash, myalgia, pruritus, arthralgia, abdominal pain, vomiting, dark urine, pale stools, and abnormal liver function [116,163,164,165]. The manifestations and outcomes of HEV infection during pregnancy are associated with the infecting genotype.

HEV-1 or HEV-2 infection poses severe risks to pregnant women. Most cases of HEV-1 and HEV-2 infection in pregnant women have been reported in resource-limited settings in Asia [16,39,40,41,166], predominantly in India [16,39,40,41] and in Africa [27,29]. The incidence of hepatitis E in pregnant women was six-fold higher than in non-pregnant women and eight-fold higher than in men [99]. HEV-1 and HEV-2 infection is associated with acute liver failure (ALF) during pregnancy [167]. Compared with men and non-pregnant women, pregnant women face a higher risk of developing ALF following HEV infection [22,99]. HEV-infected pregnant women also exhibit a higher incidence of ALF than pregnant women with other forms of viral hepatitis [22,23]. The frequency of ALF among HEV-infected pregnant women ranged from 9.1% to 69.2% in India [12,13,15,22,23] and was 14.2% in the United Arab Emirates [17]. HEV infection significantly increases the risk of maternal death [22,168]. The case fatality rate (CFR) in pregnant women was approximately 30-fold higher than in the general population [24] and approximately 10-fold higher than in non-pregnant women [39]. During the HEV outbreaks in China (1986–1988) and Niger (2017), 57.1% and 44.7% of deaths, respectively, occurred in pregnant women [165,169]. The CFR in HEV-infected pregnant women with HEV-1 and HEV-2 was 2.5–27.4% [16,27,29,39,40,41,166]. Reported CFR values vary widely across studies conducted in endemic settings where HEV-1 and HEV-2 predominate, ranging from 1.8% to 66.7% (PubMed was searched using the keywords “HEV”/“hepatitis E virus”/“hepatitis E,” “pregnant”/“pregnancy,” and “death”/“mortality”/“fatality”, with the last search conducted on 25 May 2026; studies were included if they reported original data with sufficient information available in the abstract or main text, whereas reviews and studies with insufficient data were excluded) (Table 2) [12,13,14,15,16,17,22,23,24,25,26,27,28,29,30,31,32,33,34,35,36,37,38,39,40,41,42,43,44,45,46,47,48,49,50,51,52,53,170,171,172,173]. Of note, most of these studies were conducted among symptomatic HEV-infected pregnant women and the infecting HEV genotype was not confirmed in most cases. A systematic review focusing on developing countries in South Asia and East Africa reported a median maternal CFR of 26% in symptomatic pregnant women in hospital settings [174]. Most HEV-associated maternal deaths occurred during the third trimester [14,22,33,41,42]. Maternal HEV-1 infection is also associated with severe adverse fetal and neonatal outcomes, including miscarriage, preterm birth, low birth weight, stillbirth, and intrauterine fetal death [16,39]. Similar adverse fetal and neonatal outcomes were observed in HEV-infected pregnant women in settings where HEV-1 and HEV-2 predominate [14,16,22,26,32,33,34,35,38,46,50,175]. Intrauterine fetal death occurred in 4.8–58.3% of HEV-infected cases [22,33,38,46,50,175]. Preterm delivery was observed in over 60% of HEV-infected pregnant women in India [14,16,22,32,34,38] and in 42.9% of those in Namibia [175]. Vertical transmission has been observed in 46.1% of HEV-1-infected pregnant women in India [16] and has been documented in 33.3–78.9% of HEV cases in India in which the infecting genotype was not confirmed [12,13,14,15,16], as well as in 100.0% of cases in the United Arab Emirates [17] (Table 2). Collectively, HEV-1 and HEV-2 infection during pregnancy substantially affects both maternal and fetal/neonatal health and is associated with a considerable case fatality rate.

Based on available reports, HEV-3 and HEV-4 infection during pregnancy appears to be less virulent than HEV-1 and HEV-2 infection. No maternal deaths have been reported, and ALF has been rare in pregnant women infected with HEV-3 and HEV-4, both in developed countries, including France [176, 177] and Germany [178], and in developing countries, such as China [117,179] and Vietnam [161]. In the USA and the UK where HEV-3 predominates, no maternal deaths among pregnant women with HEV infection were reported [180,181,182]. In the USA, up to 84% of HEV-infected pregnant women developed complications, including hemorrhage, coagulation defects, premature rupture of membranes, and polyhydramnios [180]. The rates of abnormal glucose tolerance and diabetes were significantly higher in HEV-infected pregnant women than in those without HEV infection [180,181]. The rate of coagulopathy was more than 30-fold higher in HEV-infected pregnant women than in non-HEV-infected pregnant women [181]. HEV-4 infection during pregnancy can also lead to adverse fetal/neonatal outcomes, including preterm birth and fetal distress [117]. In the USA where virtually all HEV infections have been attributable to HEV-3, abnormal heart rate or rhythm, growth restriction and preterm delivery were observed in the fetuses/neonates of HEV-infected pregnant women [180,181]. Furthermore, the frequency of fetal heart rate/rhythm abnormalities was significantly higher in HEV-infected pregnant women than in non-HEV-infected pregnant women [180,181]. Maternal HEV seropositivity significantly increased the risk of adverse fetal outcomes [117]. The incidence of adverse fetal outcomes in pregnant women with recent or current HEV infection was significantly higher than in pregnant women without recent or current HEV infection in China, where HEV-4 dominates [116]. Overall, the available evidence indicates that HEV-3 and HEV-4 are associated with adverse pregnancy outcomes even in the absence of reported maternal deaths.

**Table 2:** The number of cases, case fatality rate, vertical transmission rate, and genotypes of HEV among pregnant women.

First Author, Year of Publication	Countries	Cases (*n*)	Case Fatality Rate (%)	Vertical Transmission Rate (%)	Genotypes ^a^	References
Gurley et al., 2014	Bangladesh	21	19	-	-	[25]
Paul et al., 2020	Bangladesh	68	11.8	-	-	[26]
Escribà et al., 2008	Central African Republic	5	20	-	1 and 2	[27]
Goumba et al., 2010	Central African Republic	7	14.3	-	-	[28]
Spina et al., 2017	Chad	15	20	-	1e	[29]
Coursaget et al., 1998	Djibouti	3	33.3	-	-	[170]
Tsega et al., 1992	Ethiopia	19	42.1	-	-	[30]
Bonney et al., 2012	Ghana	3	66.7	-	-	[31]
Khuroo et al., 1995	India	10	30	62.5	-	[12]
Khuroo et al., 2003	India	65	≥35.4	-	-	[23]
Singh et al., 2003	India	22	63.6	50	-	[13]
Kumar et al., 2004	India	26	26.9	33.3	-	[14]
Patra et al., 2007	India	132	40.9	-	-	[22]
Khuroo et al., 2009	India	26	34.6	78.9	-	[15]
Salam et al., 2013	India	148	19.6	-	-	[32]
Shinde et al., 2014	India	52	32.7	-	-	[33]
Prasad et al., 2016	India	55	5.5	-	-	[34]
Kumar et al., 2017	India	32	21.8	-	-	[35]
Sharma et al., 2017	India	144	12.5	46.1	1a	[16]
Gautam et al., 2018	India	6	50	-	-	[36]
Kashyap et al., 2019	India	30	8		-	[37]
Singh et al., 2019	India	142	16.9	-	-	[38]
Bhatnagar et al., 2022	India	51	23.5	-	1a	[39]
Malhotra et al., 2020	India	39	2.5		1a	[40]
Karna et al., 2020	India	442	22.2	-	1	[41]
Bista et al., 2006	Nepal	15	20	-	-	[42]
Okwara et al., 2021	Nigeria	56	1.8	-	-	[43]
Yasmeen et al., 2013	Pakistan	30	29.3	-	-	[44]
Khaskheli et al., 2015	Pakistan	45	20		-	[45]
Javed et al., 2017	Pakistan	21	14.3	-	-	[46]
Aisha et al., 2024	Pakistan	105	21	-	-	[47]
Bile et al., 1994	Somalia	22	13.8	-	-	[171]
Boccia et al., 2006	Sudan	61	31.1	-	-	[48]
Ahmed et al., 2008	Sudan	8	37.5	-	-	[49]
Rayis et al., 2013	Sudan	39	28.2	-	-	[50]
CDC, 2013	Sudan	211	10.4	-	-	[51]
Elduma et al., 2014	Sudan	4	25	-	-	[52]
Albetkova et al., 2007	Turkmenistan	62	27.4		1	[166]
Teshale et al., 2010	Uganda	189	6.9	-	-	[53]
Gerbi et al., 2015	Uganda	7	0	-	-	[172]
Amanya et al., 2017	Uganda	23	65.2	-	-	[24]
Kumar et al., 2001	United Arab Emirates	28	10.7	100	-	[17]
Sharapov et al., 2009	Uzbekistan	12	8.3	-	-	[173]
Ma et al., 2021	China	21	0	-	-	[115]
Zhang et al., 2022	China	384	0	0	-	[165]
Qian et al., 2023	China	13	0	-	4	[179]
Qian et al., 2023	China	61	0	-	4	[117]
Wen et al., 2023	China	33	0	-	-	[116]
Anty et al., 2012	France	1	0	-	3f	[176]
Bouthry et al., 2018	France	2	0	-	3c	[177]
Tabatabai et al., 2014	Germany	1	0	-	3c	[178]
Hussaini et al., 1997	UK	2	0	-	-	[182]
Wasuwanich et al., 2021	USA	95	0	-	-	[180]
Wasuwanich et al., 2024	USA	226	0	-	-	[181]
Huy et al., 2021	Vietnam	22	0	-	3a	[161]

- indicates not reported. ^a^ The genotypes in the studied pregnant women.

Collectively, these data indicate that genotype-dependent differences in clinical severity are particularly important in pregnancy. The true burden of HEV-related maternal and fetal complications may be underestimated in settings where systematic surveillance is lacking.

## 5. Mechanisms Underlying Severe HEV Infection During Pregnancy

The mechanisms underlying severe liver injury due to HEV infection in pregnant women remain poorly understood. The marked genotype-dependent differences in clinical severity suggest that both host and viral determinants contribute to disease outcome. Accumulating evidence points to the involvement of viral factors, together with altered hormonal and immune status during pregnancy, in disease pathogenesis.

Epidemiological evidence strongly indicates that HEV-1 and HEV-2 are almost exclusively responsible for severe maternal morbidity and high case fatality rate associated with HEV infection in pregnant women. An ex vivo model demonstrated that HEV-1 replicated more efficiently than HEV-3, produced higher quantities of infectious progeny virions, and caused severe tissue alterations at the maternal–fetal interface [183], suggesting that genotype may significantly influence disease severity in pregnant women. HEV-1 infection impaired type III interferon secretion, sustained efficient replication, markedly increased pro-inflammatory cytokines and chemokines, and promoted tissue damage [183]. The recently discovered ORF4, which is unique to HEV-1, interacts with multiple viral and host proteins to promote HEV-1 replication [19]. HEV mutations also play a role in severe HEV infection [184,185,186]. The V27A and D29N mutations in the methyltransferase region of ORF1 were significantly associated with poor outcomes in patients with ALF, suggesting an important role in enhancing HEV replication [184]. The V239A mutation in the helicase domain of ORF1 may be associated with increased HEV virulence [185]. Two ALF-associated mutations (A317T and V1120I) significantly enhanced HEV-1 replication in vitro [186]. Pregnant women presenting with ALF and severe fetal complications exhibited substantially higher HEV RNA viral loads [187,188]. Collectively, these findings suggest that viral factors—including genotype heterogeneity, viral variants, and viral load—may play important roles in severe HEV infection during pregnancy.

As pregnancy progresses, hormone levels change dramatically, reaching concentrations higher than at any other time [189]. Estrogen and progesterone levels reach physiological peaks during the third trimester, chronologically coinciding with the highest incidence of HEV-associated ALF and maternal death. Both estrogen and progesterone have been shown to enhance HEV replication in vitro [190,191,192], with progesterone acting through nonclassical progesterone receptors [190] HEV-positive pregnant women with ALF exhibited significantly higher levels of progesterone, estrogen, and β-HCG than HEV-negative pregnant women with ALF [193]. Estrogen and ESR2β levels were significant predictors of maternal mortality in HEV-infected pregnant women [38]. Reduced expression of the progesterone receptor (PR) and progesterone-induced blocking factor (PIBF) was associated with poor pregnancy outcomes in HEV infection [187]. Progesterone receptor mutations have been identified as risk factors for HEV infection and severe disease during pregnancy [187,194]. These findings suggest that hormonal changes during pregnancy may also contribute to severe HEV infection and its adverse outcomes.

Several alterations in innate and adaptive immune responses have been observed in pregnant women with severe HEV infection. HEV-infected pregnant women with ALF showed diminished cellular immunity, characterized by lower CD4+ T cell counts and lower CD4/CD8 ratios, as well as and higher CD8+ T cell counts [193]. An altered Th1/Th2 balance, marked by an elevated IL-12/IL-10 ratio, has been associated with adverse pregnancy outcomes in HEV-infected patients [187]. HEV-infected pregnant women with ALF exhibited decreased macrophage phagocytic activity, reduced TLR3 and TLR7 expression, and weakened TLR signaling [195]. T cell activation, leukocyte cell–cell adhesion, regulation of lymphocyte activation, and immune response-regulating signaling pathways were significantly downregulated in HEV-infected pregnant women with ALF compared with those without ALF [196], suggesting suppressed immune responses in the former group. Cytokine profiles were associated with disease severity and adverse pregnancy outcomes in HEV infection during pregnancy [32,187,197,198,199,200]. HEV-infected pregnant women showed significantly higher TNF-α levels than HEV-infected non-pregnant women [32]. HEV-infected pregnant women with ALF exhibited significantly higher levels of both Th1 cytokines (TNF-α, IFN-γ, IL-2, and IL-12) and Th2 cytokines (IL-6 and IL-10) than those with AVH [187,198,199]. HEV-infected pregnant women with adverse pregnancy outcomes also showed higher levels of TNF-α, IL-6, IFN-γ, and TGF-β1 [32,198]. TNF-α and IFN-γ were significantly and positively correlated with HEV viral load [198]. A recent study showed that pregnant women with HEV-induced ALF exhibited an altered inflammatory milieu, with elevated IL-22, IL-33, and chemokines (MIP-1α, SDF-1α, and fractalkine), compared with pregnant women with HEV-induced AVH [201]. Selective suppression of p65 in the NF-κB transactivation complex may lead to dysregulated immunity and severe liver damage [202]. Collectively, the above-mentioned immune dysregulation, immune dysfunction, and cytokine imbalances may be associated with severe HEV infection and adverse pregnancy outcomes.

In summary, these findings support a multifactorial model in which viral factors, hormonal changes, and immune alterations collectively increase the risk of severe disease during pregnancy. Other factors, such as micronutrient deficiencies, may also increase the risk of HEV infection during pregnancy [203]. Direct viral invasion of the placenta and the maternal–fetal interface may contribute to adverse fetal outcomes [179,183,197,204].

## 6. Treatment

Currently, no HEV-specific treatment is available. Conventional antiviral agents, such as ribavirin (RBV), pegylated interferon-α, or a combination of these drugs, have occasionally been used as off-label treatments. However, these agents are contraindicated during pregnancy owing to their potential teratogenicity and maternal side effects, leaving no definitive antiviral option for this population. Sofosbuvir has demonstrated antiviral activity against HEV in vitro [205] but failed to eliminate HEV RNA in vivo [206,207]. The development of neutralizing monoclonal antibodies (mAbs) against HEV holds considerable promise for antibody-based therapy in high-risk populations, such as pregnant women; however, this approach requires further exploration and clinical validation [208]. There is a clear need to develop novel and specific antiviral drugs against HEV [209,210]. HEV cell culture systems and animal models are important tools for the development and evaluation of antiviral agents. However, the HEV cell culture system has significant limitations, and the currently widely used HEV animal models also have major deficiencies, including limited presentations of clinical diseases, susceptible to all genotypes, ethical concerns, high cost, and operational difficulty [211]. HEV constructs harboring bioluminescent reporters can serve as alternative approaches for the development and evaluation of new treatments [210,212,213,214]. To date, recombinant HEV constructs carrying Gaussia luciferase (Gluc) [86,209,213,214,215], nanoluciferase (NanoLuc) [216], the nanoKAZ gene [210], and the HiBiT small peptide tag [212] have been reported, and these tools are expected to facilitate the development of novel anti-HEV agents.

Management of HEV infection during pregnancy currently relies on diligent monitoring and intensive care. Most symptomatic HEV infections present as AVH, which is usually self-limiting. Thus, the primary approach focuses on symptomatic and supportive care, including medications to mitigate hepatitis, adequate rest, and appropriate nutritional intake. For cholestatic acute hepatitis, antihistamines, cholestyramine, and/or ursodeoxycholic acid may be used to relieve bile stasis. Given that HEV-infected pregnant women are at high risk of ALF and that the disease course is unpredictable, continuous evaluation is critical. This includes serial assessment of hepatic synthetic function (e.g., albumin, prothrombin time, and international normalized ratio), coagulation profile, blood glucose, renal function, and neurological status for early detection of encephalopathy. If ALF occurs, intensive supportive medical care is essential for managing major complications, including encephalopathy, cerebral edema, coagulopathy, hypoglycemia, sepsis, and renal failure. Aggressive treatment with artificial liver support systems or liver transplantation may also be considered when necessary [217,218,219]. Concurrently, diligent obstetric monitoring—including fetal heart rate, biophysical profile, and growth parameters—is required to assess the stage of pregnancy, fetal well-being, and growth. The role of termination of pregnancy in HEV-infected pregnant women with ALF remains controversial [220,221,222]. A retrospective study showed that significantly lower mortality was observed in patients who delivered the fetus and had grade I, II, or III hepatic encephalopathy [221]. A case report also described promising results following induction of labor in an HEV-infected pregnant woman with ALF [220]. However, the available evidence remains insufficient to support a firm recommendation, and some investigators do not recommend routine pregnancy termination for women with HEV-associated ALF [222,223].

In summary, the available therapeutic options for HEV infection during pregnancy remain limited and largely supportive. The absence of pregnancy-specific antiviral therapy underscores the urgent need for prospective studies evaluating both efficacy and safety in this population.

## 7. Prevention

Prevention is a crucial strategy for reducing the global disease burden of HEV infection in pregnant women. Improved sanitation, water quality, hygiene practices, and food safety are essential and lower the risk of HEV infection; however, the development of an effective HEV vaccine would provide an additional preventive approach.

The HEV capsid protein encoded by ORF2 is the primary target of HEV vaccines. Several recombinant vaccine candidates based on truncated ORF2 protein have been developed over the years. To date, only four HEV vaccine candidates—the HEV 239 vaccine [224,225,226] (Xiamen Innovax Biotech, Xiamen, China), the rHEV vaccine [227] (GlaxoSmithKline, London, UK), the ZyVac-HEV vaccine [56] (Zydus Lifesciences, Ahmedabad, India), and the HEV p179 vaccine [228] (Changchun Institute of Biological Products, Changchun, China)—have progressed from preclinical testing to human clinical trials. The first three vaccines were developed based on HEV-1 [56,224,225,226], whereas the last was developed based on HEV-4 [228]. The rHEV vaccine, administered on a three-dose schedule, demonstrated a favorable safety profile and an efficacy of 95.5% in preventing symptomatic hepatitis E [227]; however, its development was discontinued for commercial reasons [227,229]. The HEV p179 vaccine and ZyVac-HEV remain under development [56,228]. The HEV 239 vaccine (amino acids 368–606), known as Hecolin^®^, is the only HEV vaccine currently approved. Administered in a three-dose schedule, it has shown efficacies of 100.0% (95% CI: 72.1–100.0%) within 12 months [224], 93.3% (95% CI: 78.6–97.9%) over 4.5 years [225], and 86.6% (95% CI: 73.0–94.1%) over 10 years [226] in the general population. The HEV 239 vaccine has also been used successfully to control protracted outbreaks in South Sudan, with an adjusted two-dose effectiveness of 89.4% (95% CI: 56.4–98.0%) in a test-negative design [230], suggesting potential operational feasibility in emergency settings. Notably, the HEV 239 vaccine demonstrated cross-genotype protection against HEV-4 in the phase III clinical trial [224]. Analysis of memory B cell responses in volunteers who received three doses of the HEV 239 vaccine suggested that the vaccine may also confer protection against HEV-3 [231].

The HEV 239 vaccine is a promising candidate for prevention in pregnant women; however, important evidence gaps remain regarding its use during pregnancy. Owing to ethical and safety constraints, pregnant women were not included in clinical trials of the HEV 239 vaccine [224]. Consequently, the available evidence on its safety and efficacy in pregnant women is limited, and establishing its safety profile during pregnancy remains a priority [232]. Most available data concerning the safety of the HEV 239 vaccine during pregnancy are derived from post hoc analyses of clinical trials, which have yielded conflicting findings [233,234,235,236,237]. A post-hoc analysis of a cluster-randomized trial in Bangladesh showed that HEV 239 vaccination before or during pregnancy was associated with an increased risk of miscarriage [233,234]. By contrast, post-hoc analyses of two phase III clinical trials involving the HEV 239 vaccine suggested that vaccination before or during pregnancy was not associated with increased risks of miscarriage [235,236]. A recent study conducted in South Sudan, which included more than 2000 vaccinated pregnant women, provided real-world evidence suggesting that administration of the HEV 239 vaccine during pregnancy was not associated with an increased risk of fetal loss [237]. At present, the effectiveness of the HEV 239 vaccine in preventing hepatitis E during pregnancy remains unknown, because the incidence of hepatitis E was lower than expected in the phase IV trial [238]. The HEV 239 vaccine has been shown to prevent HEV-related adverse outcomes in a pregnant animal model [239]. Collectively, these findings suggest potential safety and possible benefit, but definitive conclusions regarding the vaccine’s safety and efficacy during pregnancy require further investigation. Further well-designed prospective studies in pregnant women are needed to define the vaccine’s safety and efficacy profile. To address these gaps, a phase II, randomized, observer-blind, placebo-controlled trial was launched in 2024 in Pakistan to evaluate the safety and immunogenicity of the HEV 239 vaccine in healthy pregnant women [NCT05808166] [56].

## 8. Conclusions

HEV infection remains a substantial threat to maternal and fetal health worldwide. Current evidence indicates that HEV-1 and HEV-2 infections are more strongly associated with severe disease and adverse pregnancy outcomes, whereas HEV-3 and HEV-4 appear to be less frequently linked to maternal mortality but may still contribute to adverse pregnancy outcomes. Multiple factors—including viral determinants, immune alterations, and hormonal changes—may be involved in the pathogenesis of severe HEV infection in pregnant women. However, the mechanisms underlying severe HEV infection during pregnancy remain incompletely understood and warrant further in-depth investigation. Genotype characterization during pregnancy is important, yet such information is unavailable in most prevalence studies conducted in this population, even though it may inform the development of intervention and management guidelines for HEV infection during pregnancy. The HEV 239 vaccine holds promise as a tool for controlling HEV infection in pregnant women, with current data suggesting an acceptable safety profile during pregnancy; however, the available evidence remains limited, and additional clinical studies are urgently needed to provide definitive evidence of its safety and efficacy in this population. Furthermore, the importance of developing effective treatments for HEV infection in pregnant women should be emphasized. An improved understanding of these issues will facilitate the development of more effective prevention and management strategies for HEV infection during pregnancy.

## Data Availability

No new data were created or analyzed in this study. Data sharing is not applicable to this article.

## Abbreviations

ALF	acute liver failure
AVH	acute viral hepatitis
CFR	case fatality rate
CMV	cytomegalovirus
EBV	Epstein–Barr virus
ELISA	enzyme-linked immunosorbent assays
HAV	hepatitis A virus
HBV	hepatitis B virus
HCV	hepatitis C virus
HEV	Hepatitis E virus
HEV-1	HEV genotype 1
HEV-2	HEV genotype 2
HEV-3	HEV genotype 3
HEV-4	HEV genotype 4
LAMP	loop-mediated isothermal amplification
mAbs	monoclonal antibodies
ORFs	Open reading frames
ORF2^S^	the free secreted form of the ORF2 protein
PIBF	Progesterone-induced blocking factor
PR	Progesterone receptor
RBV	ribavirin
RPA	recombinase polymerase amplification
RT-PCR	reverse transcription-polymerase chain reaction
WHO	World Health Organization
